# Effect of Opioid on Adult Hippocampal Neurogenesis

**DOI:** 10.1155/2016/2601264

**Published:** 2016-04-05

**Authors:** Yue Zhang, Horace H. Loh, Ping-Yee Law

**Affiliations:** Department of Pharmacology, University of Minnesota, 6-120 Jackson Hall, 321 Church Street SE, Minneapolis, MN 55455-0217, USA

## Abstract

During the past decade, the study of the mechanisms and functional implications of adult neurogenesis has significantly progressed. Many studies focus on the factors that regulate proliferation and fate determination of adult neural stem/progenitor cells, including addictive drugs such as opioid. Here, we review the most recent works on opiate drugs' effect on different developmental stages of adult hippocampal neurogenesis, as well as the possible underlying mechanisms. We conclude that opiate drugs in general cause a loss of newly born neural progenitors in the subgranular zone of dentate gyrus, by either modulating proliferation or interfering with differentiation and maturation. We also discuss the consequent impact of regulation of adult neurogenesis in animal's opioid addiction behavior. We further look into the future directions in studying the convergence between the adult neurogenesis field and opioid addiction field, since the adult-born granular cells were shown to play a role in neuroplasticity and may help to reduce the vulnerability to drug craving and relapse.

## 1. Introduction

During the past two decades, it has been well established that new neurons were born continuously throughout life in the brains of many species, including human [1, 2]. In normal conditions, adult neurogenesis appears to be restricted in two discrete brain regions: the subventricular zone (SVZ) of the lateral ventricle [3] and the subgranular zone (SGZ) of the hippocampal dentate gyrus (DG) [4]. Since then, substantial research has been made to study the intrinsic and extrinsic factors that regulate adult hippocampal neurogenesis, for newborn neurons in the SGZ could contribute to specific hippocampal functions such as spatial learning, pattern discrimination, and mood regulation [5, 6]. Several classes of neural stimulants have been shown to alter adult neurogenesis, including addictive drugs such as methamphetamine [7], cocaine [8], and opioid [9].

Opiate drugs are powerful analgesics which are also among most commonly abused addictive drugs. They can cause long-lasting changes in the brain, which influence many different forms of neural plasticity, such as the stability of dendritic spines [10] and long-term potentiation [11]. Adult hippocampal neurogenesis is also among forms of neural plasticity mechanism regulated by opiates. However, the effects of opiate on hippocampal neural progenitors are controversial in many cases and are largely dependent on the manner in which the drug was administered [12]. Also, since adult neurogenesis is a long and continuous progress which consists of a series of developmental events, opiate drugs could exert their action on multiple types and stages of the neural stem/progenitor cells (NSPCs).

The proliferation, differentiation, and maturation of adult-born granular cells (GCs) are controlled by a series of genetically programmed fate choices [13], and NSPCs in adult hippocampus could be divided into several types according to their different developmental stages. For instance, radial-glia-like stem cells, which express glial fibrillary acidic protein (GFAP) and nestin and have several other astrocytic features, are defined as Type-1 cells [14]. Type-2 cells are oval-shaped, highly proliferative cells with short processes which express nestin but not GFAP [15]. Type-3 cells are neuroblasts which express doublecortin (DCX) and polysialylated form of the neural cell adhesion molecule (PSA-NCAM) [16]. Different opiate drugs may target any of these cell types mentioned above, either directly or indirectly. Here, we summarize the most recent works correlated with opiates' effect on regulating proliferation, differentiation, or survival of adult-born hippocampal GCs (Table 1).

## 2. Opioid Modulates Adult Neural Progenitors Proliferation

The most traditional and commonly used method to detect the proliferating cells in adult brain is by using exogenous markers of DNA synthesis, such as thymidine analog bromodeoxyuridine (BrdU), to label and track the birth of new born cells [31, 32]. The first report connecting opioid and adult neurogenesis was in 2000. Eisch et al. showed that chronic morphine, administered via subcutaneous pellet, decreased the number of proliferating cells labeled with BrdU in the SGZ in rodents; similar effect was also observed in rats after chronic self-administration of heroin [9]. Since then, evidences were accumulated from both sides to established opiate's negative impact on proliferation of adult-born GCs (Table 1). For instance, proliferating cells in SGZ marked by two endogenous cell cycle markers, proliferating cell nuclear antigen (PCNA) and phosphorylated histone H3 (pHisH3), are largely reduced by chronic morphine, and triple labeling for BrdU, PCNA, and pHisH3 revealed that morphine-treated mice have a shorter Gap2/mitosis (G(2)/M) phase [20]. Rats injected with morphine sulfate (20 mg/kg) daily for 1 week were shown to have a strong reduction of cellular proliferation marked by fewer cells immunoreactive (IR) for PSA-NCAM, a cell surface protein that is transiently expressed by newly generated neurons during development. Such reduction was followed by a rebound increase after 1-week withdrawal and a return to normal after 2-week withdrawal [21]. It was demonstrated that morphine pellet implantation for 24–96 hours decreased the proliferating cells labeled by BrdU and cycle marker Ki67 in DG [22]. Other opiate analgesics like buprenorphine, administered via subcutaneous injections (0.05 mg/kg) over a 3-day period in mice, also decreased the number of actively proliferating 5-iodo-2-deoxyuridine (IdU) labeled cells [26], while no such effect was observed with synthetic opiate methadone [27]. Meanwhile, knock-out of mu-opioid receptor, on the contrary, was shown to enhance ischemia-induced generation of immature hippocampal neurons [33]. Following extinction from heroin-seeking behavior, the formation of immature neurons in the DG was increased, represented by DCX-IR cells [25]. In addition, there are also reports which suggest that chronic morphine treatment influences neurogenic microenvironment in DG by regulating certain growth factors, such as increasing the pro-proliferative factor and vascular endothelial growth factor (VEGF) [34].

However, opiate's effect on adult neurogenesis seems to be dependent on the paradigm of the experiment design, such as test in vitro or in vivo, and drug administration paradigm. In isolated rat hippocampal neural progenitor cells, incubation with *β*-endorphin for 48 h increased the total DNA content and the number of cells expressed of PCNA and pHisH3. This proliferative effect was antagonized by naloxone [17]. The same group also reported that mu- and delta-opioid receptor (MOR and DOR) antagonists decrease proliferation of cultured neural progenitor cells [18]. Similarly, a longer acting opioid antagonist naltrexone was shown to decrease cellular proliferation in the adult rat hippocampus [19]. These results are conflicting with more recent observations, which showed cultured mouse hippocampal neural progenitor cells treated with morphine for 24 h demonstrated decreased BrdU expression in a dose dependent manner [29]. This discrepancy in morphine's effect on neural proliferation remains within in vivo experiments, in which implantation of morphine pellets resulted in negative effect on adult neurogenesis [9, 22], while intraperitoneally injection of escalating dose of morphine failed to show any significant influence [12]. Such inconsistency of morphine's effect may be due to difference in blood levels of morphine; when implanted with morphine pellet, the drug level in blood is relevantly stable and caused decrease in number of proliferating cells, whereas the injection paradigms that produced transient spikes in drug blood levels fail to produce significant effect on hippocampal neural proliferation [12]. Nevertheless, studies in our lab support the assumption that opiate negatively regulated neural proliferation, for morphine daily injection in a condition place preferences (CPP) paradigm, decreased the number of neural progenitors in DG labeled by DCX and other neurogenesis markers in mice [28]. Further research in this model revealed that such reduction may be due to morphine's effect in modulating neural progenitors' differentiation, rather than regulating proliferation, which will be discussed in detail in the following section.

## 3. Opioid Modulates Adult Neural Progenitors Differentiation and Maturation

The radial-glia-like neural stem cells in SGZ went through asymmetric cell division and gave rise to different types of progeny, including progenitors retained self-renew capability, neuroblasts, astrocytes, and oligodendrocytes [35, 36]. A growing body of literature indicated that opiate drugs not only influence hippocampal GCs' proliferation, but also interfered with differentiation and future development process. In adult rat hippocampus, repeated morphine treatment altered the GABAergic phenotype of adult hippocampal GCs by significantly increasing the mRNA transcription of glutamate decarboxylase-67, a GABA synthesizing enzyme [21]. By examining the costaining of BrdU and cell cycle marker ki67 in mouse SGZ, it was found that morphine treatment increases the percent of BrdU-IR cells that were type 2b and decreased the percent of BrdU-IR cells that were immature neurons [22]. Analysis of the double-labeled cells in cultured mouse hippocampal progenitors treated with morphine showed a decrease in cells costained for BrdU with nestin and an increase in cells costained with BrdU and neuron-specific class III beta-tubulin (TUJ1) compared to cells treated with saline [29]. Incubation of adult hippocampal progenitors with endogenous opioid peptide beta-endorphin resulted in a threefold increase in oligodendrogenesis but no significant change in astrogliogenesis [37]. Although having some discordance in conclusions, these observations indicated that opioid could play a role in regulating adult hippocampal neural differentiation and maturation.

Recent study in our lab interpreted in detail that morphine exposure affects hippocampal neurogenesis by modulating cell-lineage in isolated hippocampal progenitor cells. In cultured NSPCs, morphine treatment activates MOR and downstream signaling pathways, including extracellular signal-regulated kinase (ERK) activation [38]. Phosphorylated ERK in cytosol is capable of phosphorylating TAR RNA-binding protein (TRBP), a cofactor of Dicer, and the Dicer activity enhancement promotes the maturation of miR-181a. This drives downregulation of Prospero homeobox protein 1 (Prox1) and an upregulation of Notch1 expression, while the Notch1 signaling plays an important role in regulating cell fate of the adult-born hippocampal GCs [39, 40]. Thus, morphine favors the progenitor cells differentiation into glia instead of neuron by regulating Prox1/Notch1 activities via its control of miR-181a level [41]. Another opiate drug fentanyl did not show such effect, since fentanyl activated ERK via a *β*-arrestin-dependent pathway, and the activated ERK translocates to the nucleus [42].

Furthermore, the activity of a transcriptional factor, neurogenic differentiation 1 (NeuroD1), was also shown to be regulated by morphine treatment [43]. NeuroD1 is a basic helix-loop-helix transcription factor that is expressed during glutamatergic neurogenesis in the developing cerebellum and in the adult hippocampal DG [13, 44]. It was shown to be involved in the differentiation of the progenitor cells and migration of immature neurons in the dentate gyrus [45]. Several in vivo studies support the fact that NeuroD1 has an important role in neuronal fate determination during both embryonic and adult neurogenesis [46, 47] and is essential for the survival and maturation of adult-born neurons [48]. Thus, by negatively regulating NeuroD1 activity, morphine impaired the differentiation of newborn GCs, leading to a reduction in neuroblasts and immature neurons expressing DCX and TUJ1.

## 4. Opioid Modulates Adult Neural Progenitors Survival and Apoptosis

After being generated by neural stem cells in the DG of hippocampus, a large portion of these progenitors die within a few days following their birth [49]. It is reasonable to assume that the neural precursors which fail to differentiate into functional immature neurons would go through apoptosis. The massive cell death of adult-born granular neurons may serve as a natural selective mechanism since it has been demonstrated that cell survival and death are both important during learning and memory [50]. Whether opiates interfere with this process remains to be demonstrated.

Chronic morphine and heroin treatment was shown to decrease GCs survival in vivo, by largely decreasing the number of 4-week-old BrdU-labeled cells in the granule layer of the DG in drug-group rats compared to control rats [9]. However, direct evidence of opiate drugs inducing apoptosis of adult hippocampal progenitors is deficient and inconsistent. Chronic morphine transiently increases cell death in the SGZ of mice, for the activated caspase-3 cell counts were increased after 24 but not 96 h [22]. Morphine exposure in cultured NSPCs led to a significant increase in caspase-3 activity in the nestin and GFAP positive cells, but not in TUJ1 positive neurons [29, 51]. Knock-out of mu-opioid receptor, in the contrary, was shown to enhance adult-born hippocampal GCs' survival, suggesting endogenous opioid has a negative effect on adult hippocampal neurogenesis [52]. However, minimal buprenorphine treatment was shown to increase the survival of newly born cells in mice DG of hippocampus [26]. In other cases, opioid drugs such as morphine were not associated with hippocampal neural apoptosis [53].

**Figure 1:**
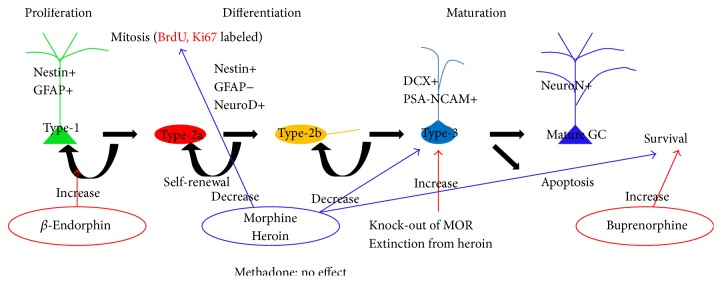
Different types of opioids act on different stages of adult neurogenesis in the DG (with expression pattern of specific markers).

Overall, we summarize that the effects of opioid on NSPCs may vary among different drugs and experimental methods, and one opioid receptor agonist may act on multiple stages of NSPCs, including proliferation, differentiation, and survival (Figure 1).

## 5. Adult Neurogenesis Regulation Correlates with Opioid Addiction 

During development, newborn neurons in the adult SGZ migrate into the granule cell layer of the dentate gyrus and integrate into existing hippocampal circuit [54, 55]. The immature neurons have higher input resistance, more depolarized resting membrane potentials, and small, broad action potentials compared to mature neurons [56], so they were more flexible in transition of neural plasticity and may have substantial roles in hippocampus function during learning and memory [57, 58]. Since hippocampus has been implicated in drug reward and relapse [59, 60], recent studies suggested that adult neurogenesis in DG of hippocampus also has substantial roles in opiate drug addiction cycle. For instance, suppression of adult neurogenesis by long-term stress had significant positive relationships with ratings of craving for heroin [61]. Some positive regulators of hippocampal neurogenesis like environment enrichment and voluntary exercise, on the contrary, prevent the development of morphine induced CPP [62, 63], decreased the rewarding effect of heroin [64], and maintained heroin self-administration [65]. These results suggest a negative correlation between opiate drug addiction and level of adult hippocampal neurogenesis.

## 6. Conclusion and Prospects

So far, accumulating evidences have demonstrated that multiple opiate drugs interfered with proliferation, differentiation, maturation, and survival of developing adult-born hippocampal neural precursors. These studies represent that most of the opiates have an adverse effect on adult hippocampal neurogenesis, by decreasing the total number of proliferating cells and cells survival in the SGZ of DG area, but there are also some exemptions. For neural differentiation, opiate such as morphine is likely to impede early progenitors which differentiate into neuroblasts but favor the differentiation into glia.

The detailed mechanism of such regulation on hippocampal neurogenesis of opiates remains to be clarified. There are assumptions of opiate directly acting on neural progenitors with MOR and DOR on the cell surface [17, 18] and also evidences that opiate modulates the neurogenic microenvironment of the DG, to indirectly influence the cell proliferation by growth factors in the hippocampus [23]. An alternative explanation is that progenitors of certain stage (3–7 days after birth) start to form dendrites, which receive neurotransmitters from intermediate neurons [66]. It has been reported that immature neurons (14–28-day postmitotic) are not inhibited but excited by GABAergic activity [54]. Also, exposure to novel environments increases GABAergic tone in the DG and facilitates the generation of LTP [58]. Thus, a possible mechanism for regulation of adult hippocampal neurogenesis is that early neural progenitors need exciting signals from existing circuit for future differentiation and maturation, and opioid agonists interfere with this process by decreasing GABA release in the interneuron [67, 68]. It is intriguing to further investigate these possible mechanisms and to determine whether morphine exhibits its effect directly or indirectly on neural progenitors in the SGZ of hippocampus.

Current studies also indicate that opiates' rewarding effect and drug associate memory are related with the manipulation of adult hippocampal neurogenesis. When hippocampal neurogenesis is enhanced by physical excise or environment enrichment, the animals show a lower response to drug craving and reward [62–65]. When overexpressing NeuroD1 in dentate gyrus to induce neural differentiation, the animal shows much longer memory of their drug experience, represented by condition place preference (CPP) extinction time; when knocking down NeuroD with RNA interference method it has an opposite effect [28]. These results suggest that the progenitors at certain stage of development may serve as key players during memory formation of drug experience associated with environmental cues. In summary, current studies suggest that opiates are involved in the proliferation and fate determination of adult-born GCs in the SGZ of hippocampus, and the manipulation of adult hippocampal neurogenesis in return influences rewarding effect and drug-experience memory that associate with the opioid addition. These studies provide a creative aspect to examine the subject of adult neurogenesis' contribution to opioid addiction.

## Figures and Tables

**Table 1:** Effects of drugs on different stages of adult neurogenesis.

Drugs	Species	Administration paradigm	Effects			References
			Proliferation	Neural differentiation	Survival	
Morphine	Rat	Acute injection	—		—	[9]
Morphine	Rat	Pellet implantation	↓		↓	[9]
Heroin	Rat	Self-administration	↓		↓	[9]
*β*-Endorphin	Rat	In vitro, chronic	↑			[17]
# naloxone	Rat	In vitro, chronic	↓	↑		[18]
# naltrindole	Rat	In vitro, chronic	↓			[18]
# naltrexone	Rat	Acute injection	↓			[19]
Morphine	Mouse	Pellet implantation	↓			[20]
Morphine	Rat	Multiple injections	↓			[21]
Morphine	Mouse	Pellet implantation	↓	↓		[12, 22, 23]
Morphine	Mouse	Multiple injections	—			[12]
Met-enkephalin	Zebra finch	In vitro, chronic	↓			[24]
# naloxone	Zebra finch	In vitro, chronic	↑			[24]
		In vivo, chronic				
Heroin	Rat	Extinction of self-administration		↑		[25]
Buprenorphine	Mouse	Multiple injections	↓		↑	[26]
Methadone	Rat	Multiple injections	—	—	—	[27]
Morphine	Mouse	Multiple injections	—	↓		[28]
Fentanyl	Mouse	Multiple injections	↑	—		[28]
Morphine	Mouse	In vitro, chronic	↓		↓	[29]
Morphine	Mouse	Multiple injections		↓		[30]

↑, upregulation; ↓, downregulation; —, no significant differences; #, opioid receptor antagonist.

## Abbreviations

BrdU: 5-Bromo-2-deoxyuridine
CPP: Conditioned place preference
DCX: Doublecortin
DG: Dentate gyrus
DOR: Delta-opioid receptor
ERK: Extracellular signal-regulated kinase
GCs: Granular cells
GFAP: Glial fibrillary acidic protein
MOR: Mu-opioid receptor
NeuroD1: Neurogenic differentiation 1
NSPC: Neural stem/progenitor cells
IR: Immunoreactive
PCNA: Proliferating cell nuclear antigen
pHisH3: Phosphorylated histone H3
Prox1: Prospero homeobox protein 1
PSA-NCAM: Polysialylated form of the neural cell adhesion molecule
SGZ: Subgranular zone
SVZ: Subventricle zone
TRBP: TAR RNA-binding protein
TUJ1: *β*-III tubulin
VEGF: Vascular endothelial growth factor.
